# Determination of the Prevalence of Knee and Hip Clinical Osteoarthritis in the Active Professional Male Footballer and Its Association with Pain, Function, Injury and Surgery

**DOI:** 10.3390/sports11070136

**Published:** 2023-07-18

**Authors:** Lervasen Pillay, Dina C. Janse van Rensburg, Gopika Ramkilawon, Mario Maas, Emmanuel Orhant, Jussi Rantanen, Jari Salo, Gino Kerkhoffs, Vincent Gouttebarge

**Affiliations:** 1 Amsterdam UMC location University of Amsterdam, Department of Orthopedic Surgery and Sports Medicine, Meibergdreef 9, 1105 Amsterdam, The Netherlands; drpillay@absamail.co.za (L.P.);; 2 Section Sports Medicine, Faculty of Health Sciences, University of Pretoria, Pretoria 0028, South Africa; 3 Department of Statistics, University of Pretoria, Pretoria 0028, South Africa; 4 Amsterdam UMC location University of Amsterdam, Department of Radiology and Nuclear Medicine, Meibergdreef 9, 1105 Amsterdam, The Netherlands; 5 Academic Center for Evidence-Based Sports Medicine (ACES), 1105 Amsterdam, The Netherlands; 6 Amsterdam Movement Sciences, Aging & Vitality, Musculoskeletal Health, Sports, 1105 Amsterdam, The Netherlands; 7 Amsterdam Collaboration on Health & Safety in Sports (ACHSS), IOC Research Center of Excellence, 1105 Amsterdam, The Netherlands; 8 French Football Federation (FFF), Clairefontaine Medical Centre, FIFA Medical Center of Excellence, 93216 Clairefontaine, France; 9 Orthopaedics and Sports Clinic, Mehilainen NEO Hospital, 20520 Turku, Finland; 10 Sports Hospital Mehilainen, 41400 Helsinki, Finland; 11 Football Players Worldwide (FIFPRO), 2132 Hoofddorp, The Netherlands

**Keywords:** active football player, knee osteoarthritis, hip osteoarthritis, function osteoarthritis, pain osteoarthritis

## Abstract

Objective: To comment on and explore (1) the prevalence of clinical knee and hip osteoarthritis (OA); (2) the association between pain or function and clinical knee or hip OA; (3) the association between injury or surgery and clinical knee or hip OA. Methods: Participants were recruited from FIFPRO members. A total of 101 footballers consented to answer (1) a developed questionnaire, (2) patient-reported outcome measures, and (3) be evaluated by their team physician for clinical knee or hip OA. Results: Of the 53% evaluated for clinical knee and hip OA, a prevalence of 9.43% and 7.55% of knee and hip OA, respectively, was found. There was a significant and strong association between knee (*p* = 0.033; Cramers v Value = 0.523) and hip pain (*p* = 0.005; Cramers v Value = 0.602) and clinical OA. A significant association existed between Hip dysfunction and Osteoarthritis Outcome short form Scores and clinical OA of the hip (*p* = 0.036). The odds of clinical knee OA were 1.5 and 4.5 times more after one or more injuries or surgeries, respectively. There was no association between playing position and clinical OA. Conclusion: There is a low prevalence of a clinical knee or hip OA in the active professional male footballer. Pain may be a valid symptom to predict or monitor knee or hip OA. Validated assessment tools should be utilised to identify a negative effect on function. The odds of developing clinical OA in the knee with the number of injuries or surgeries. The hip presents with earlier clinical signs of OA compared to the knee.

## 1. Introduction

Football is the most popular global sport, with most countries having a professional league. According to the Professional Football Report of the Federation Internationale de Football Association (FIFA), in 2019, 128,983 professional players were registered across 187 countries [1]. Epidemiological evidence has found that these professional footballers have a high risk for injury. The Union of European Football Associations (UEFA) injury surveillance study evaluated 3302 players over nearly 20 years and found a significant loss of game time due to injuries [2]. Injuries can be sustained through contact and non-contact mechanisms (e.g., Anterior Cruciate Ligament rupture) and may involve muscle, bone, ligament, tendon, and cartilage [3].

Research has found that the knee joint is the most common football-related injured region among professional footballers in South Africa, Germany, and the United Kingdom [4,5,6]. Predisposing factors such as previous injuries [7], high workload [2], surface type, and poor pelvic control increase the risk of injury [8,9]. It has been proposed that football-specific movements [10] may increase the risk of lower limb joint-related trauma and that excessive joint load contributes to earlier joint degeneration compared to the general population [11]. Retired professional footballers are more likely to experience knee pain compared to the general population when adjusted for age [11].

Osteoarthritis (OA) following joint trauma is termed Post Traumatic Osteoarthritis (PTOA). Several studies have observed that footballers develop knee and hip osteoarthritis later on in their active playing years and early on after retirement. Most would require surgical intervention at some stage [11,12,13,14,15,16,17,18,19,20]. Most of these studies diagnose OA based on radiological criteria (e.g., the Kellgren–Lawrence criteria) [21]. Previous injuries have been found to be the most common predictors of the development of PTOA in the knee and hip joints [16,17,18,19]. The available literature has found that players who sustain ACL injuries and are managed by reconstruction surgery can still develop knee PTOA compared to those managed conservatively [19]. Previous research has also found that retired professional footballers are twice as likely to develop PTOA with every severe knee injury sustained or surgery performed. In contrast, only half the number of active professional footballers have developed knee PTOA after sustaining injuries or being subjected to surgery [13]. If one considers the hip joint and PTOA, arthroscopic hip procedures usually lead to short- and medium-term symptom improvement. However, there is inconclusive data on the long-term outcomes after these procedures [20].

Only a few studies describe the prevalence of clinical knee or hip OA in the active professional footballer and its potential association with injury and surgery. Furthermore, the available literature fails to comment on the association between function and pain and knee or hip clinical OA in the active professional footballer. The literature focuses mainly on retired professional footballers [13]. Therefore, this study aimed to comment on and explore the following in the active professional male footballer: (1) the prevalence of clinical knee and hip OA; (2) the association between pain or function and clinical knee or hip OA; (3) the association between injury or surgery and clinical knee or hip OA.

## 2. Materials and Methods

### 2.1. Study Design

An observational cross-sectional design utilising the Strengthening the Reporting of Observational Studies in Epidemiology statement to guarantee the quality of reporting [22]. The Medical Ethics Review Committee of the Amsterdam University Medical Centers (Amsterdam UMC, location AMC) provided ethical approval for the study (Drake Football Study: NL69852.018.19|W19_171#B202169). The study was conducted in accordance with the Declaration of Helsinki (2013) [23].

### 2.2. Participants and Sample Size

Participants consisted of active male professional footballers recruited by Football Players Worldwide (FIFPRO) and affiliated national unions. At least 50 participants were required to reach a power of 80% (confidence interval (CI) of 95% and an absolute precision of 6%) under the assumption of an anticipated population proportion (prevalence) of 5% [24]. The inclusion criteria for the participants were (1) a professional male footballer; (2) male; (3) able to read and comprehend texts in Dutch, English, or French; (4) between the age of 24 and 30 years. We did not include the training intensity, level of activity, and match frequency as part of the inclusion criteria nor as a variable, as these are professional male footballers and these aspects are generally higher than the general population. For this study, the definition of a professional footballer has been described by Gouttebarge et al. (2018) [13] previously: (1) trains to improve performance on the field of play; (2) competes in the highest or second highest national league level; (3) has football training and competition as major activity (way of living) or focus of personal interest, devoting several hours (generally considered as at least four hours of daily training at different levels of intensity and at least one competitive match a week of 90 min or more which may involve several hours of match-related preparation and travel) in all or most of the days for these activities and exceeding the time allocated to other types of professions or leisure activities.

### 2.3. Independent Variables Definition and Evaluation

These variables consisted of (1) pain in the knee or hip; (2) the knee injury and osteoarthritis outcome score physical function short form (KOOS-PS); (3) hip dysfunction and osteoarthritis outcome score short form (HOOS-PS); (4) a history of knee or hip injury or surgery.

Pain in the knee and hip was assessed through two questions (e.g., “How often do you experience hip pain?”) scored on a 5-point scale (from 0 “Never” to 4 “Always”). The developed questionnaire is available in Supplementary File SA.

The function was assessed using validated tools. The KOOS-PS was used to assess the level of knee function (Supplementary File SB) [25]. The KOOS-PS has seven items, each measured on a 5-point scale (from 0 to 4) and converted to a total score ranging from 0 to 100, where 0 represents total knee disability and 100 perfect knee function. The HOOS-PS was used to assess the level of hip function (Supplementary File SC) [26]. The five items are measured on a 5-point scale (from 0 to 4) and to a total score ranging from 0 to 100, where 0 represents total hip disability, and 100 represents perfect hip health. KOOS-PS and HOOS-PS have been validated in several study populations [25,26].

A history of knee or hip injury or surgery were examined through eight questions (e.g., “How many severe injuries in your left knee have you had so far as a professional footballer?”). The developed questionnaire is available in Supplementary File SA. For these questions, participants were requested to consult either their medical records (should they have them) or their team physician. For this study, a severe injury was defined as an injury that involved the knee and/or hip, which occurred during team activities (training or match) and led to either training or match absence for more than 28 days [27]. The definition is partly in line with the latest consensus statement on recording and reporting injury epidemiology in football as we have been (1) anatomically specific, but (2) we have used a different number of days to define injury severity [28]. We could not adjust the data to align completely with the consensus statement because the data was already collected before the publication of the consensus statement. We relied on the literature at the time to define data collection in football research [27].

### 2.4. Dependent Variables Definition and Evaluation

The dependent variables included the presence of clinical knee or hip OA. The team physician assessed for the presence of clinical OA in knees and hips based on both history and physical examination according to the adapted National Institute for Health and Care Excellence (NICE) criteria [29]. Osteoarthritis was diagnosed in cases of (1) activity-related joint pain; (2) restricted range of motion (ROM) of the joint; (3) either no morning joint-related stiffness or morning stiffness that lasts no longer than 30 min.

### 2.5. Descriptive Variables

Several aspects of the participants’ characteristics were collected (single questions), including, amongst others, age, weight, height, number of seasons as a professional football player, use of pain medication, career level, playing position, level of education, and the number of hours spent studying. We also collected additional information on self-reported global physical health by using the Patient-Reported Outcomes Measurement Information System Global Health form (PROMIS-GH) [30] (Supplementary File SD). This validated tool uses 10 questions to measure physical and mental health. Questions were assessed on a scale from 1 “Poor/Not at all/Always/Very severe” to 5 “Excellent/Completely/Never/None”. Only pain is assessed from 0, “No Pain”, to 10 “, Worst pain imaginable”. These values are then recorded and expressed from 0 “Poor” to 100 “Excellent”. All these descriptive variables are available in the developed questionnaire in Supplementary File SA.

### 2.6. Procedures

FIFPRO and their affiliated national unions emailed information about the study to potential participants. All participants gave informed consent electronically and completed an electronic questionnaire available in Dutch, English, or French. The questionnaire included all independent and descriptive variables. Participants were then assessed by their team physician for clinical knee or hip OA. This information accounted for the dependent variables. The questionnaires and clinical evaluation were completed and uploaded electronically using the Castor Electronic Data Capture System by Ciwit B.V. (Amsterdam, The Netherlands). This ensured capturing and integrating data collection in a private, confidential, and compliant manner. The data were further coded and anonymised to further ensure privacy and confidentiality. All uploaded information was saved on a secured electronic server that only the principal researcher could access. Participation in the study was voluntary and there was no reward for participation.

### 2.7. Statistical Analysis

Statistical analysis was performed using R software (version 4.1.3; http://www.r-project.org/ (accessed on 8 July 2023)). Descriptive analyses (mean, standard deviation [SD], frequency, and range) were performed for all variables. Body mass index was also calculated by dividing weight by the square of height in meters [31]. For our first aim, point prevalence was calculated to evaluate the prevalence of clinical knee and hip OA using a Wald-adjusted confidence interval (CI) of 95% [24]. We expressed the frequency of those with injuries or surgeries and clinical OA, as a percentage of the number of players that were clinically assessed for knee or hip OA. For our second aim, the statistical significance of the association was calculated using Fisher’s exact test (where there was a variable count of less than 5) and the Chi-squared test (where there was a variable count of at least 5). Significance was expressed as a “*p*” value (where *p* < 0.050 denoted a statistically significant association). The strength of the association was reported using Cramer’s V value, where 0 was “no strength of association” and 1 was “a perfect association”. A CI of 95% was used. For our third aim, we used Fisher’s exact test or Chi-squared test, where applicable, to calculate the statistical significance of the associations. Using the cross-tabulation method, odds ratios (OR) were calculated to report injuries or surgeries and the odds of developing clinical knee or hip OA.

## 3. Results

### 3.1. Characteristics of Participants

A total of 101 participants were included in this study. Most played as defenders, with more than half playing at a national level (Table 1).

### 3.2. Prevalence of Clinical Knee and Hip OA

Only 53% (n = 53) were assessed by their team physicians for clinical knee and hip OA. The prevalence of knee and hip OA was 9.43% (95% CI; 1.56–17.30) and 7.55% (95% CI; 0.44–14.66), respectively (Table 2).

### 3.3. Association between Pain and Function and Clinical Knee and Hip OA

Of the 101 participants, 55 participants experienced knee pain. Of the 53 participants evaluated by their team physician for clinical knee OA, only 27 experienced knee pain. Four of them had clinical knee OA with pain (14.81%). There was a significant and moderate-to-strong association between knee pain (95% CI; *p* = 0.033; Cramer’s V value = 0.523) and clinical knee OA. The total KOOS-PS score was 89, and a non-significant association between KOOS-PS and clinical knee OA (95% CI; *p* = 0.056) was calculated (Table 2).

Of the 101 participants, 33 participants experienced hip pain. Of the 53 participants evaluated by their team physician for clinical hip OA, only 17 experienced hip pain. Of these, four had clinical hip OA (23.53%). There was a significant and strong association between hip pain and clinical hip OA (95% CI; *p* = 0.005; Cramer’s V value = 0.602). There was a significant and strong association between HOOS-PS (score = 97; 95% CI; *p* = 0.005; Cramer V value = 0.602) and clinical hip OA (Table 2).

### 3.4. Association between Injury and Surgery and Clinical Knee and Hip OA

The odds of a player with a knee injury having clinical knee OA were 1.49 times more than a player without injury (95% CI; 0.16–19.32; *p* = 0.706). The odds of a player with knee surgery having clinical knee OA were 4.348 times more than a player without surgery (95% CI; 0.44–57.87; *p* = 0.150) (Table 3).

The odds of a player with a hip injury having clinical hip OA were 0.924 times more than a player with no injury (95% CI; 1.34–12.75; *p* = 0.999). The odds of a player with hip surgery could not be calculated due to zero counts in the frequency table (Table 3).

## 4. Discussion

We found a low prevalence of clinical knee and hip OA amongst active professional male football players. There were findings of a significant association between knee or hip pain and clinical knee or hip OA, between hip function and clinical hip OA but not between knee function and clinical knee OA. However, we calculated that as the number of knee injuries or surgeries increased, the odds of developing clinical knee OA increased. We did not find these odds with hip injuries or surgeries.

### 4.1. Prevalence of Knee and Hip OA

The literature shows a high incidence of knee or hip OA in retired professional male football players [11,12,15,16]. A study comparing the prevalence of knee OA in active to retired male football players concluded that the presence of knee OA is less in active (13%) than in retired (28%) players [13]. Another study (involving active and retired professional male footballers with a focus on hip OA) found a low prevalence of clinical hip OA in active (2%) and retired (8%) footballers [32]. The findings in the retired professional male footballer may be due to several factors: (1) a reduction in physical activity upon retirement; (2) a change in eating and lifestyle habits leading to weight gain and an increase in BMI; (3) having sustained more cumulative injuries, surgeries or joint injections in the past; (4) older age. We found a low prevalence of both clinical knee (9.43%) and hip OA (7.55%) in active professional male footballers. Potential explanations for our findings may be due to (1) an increased level of physical activity which includes strength and proprioception exercises; (2) younger age; (3) sustaining fewer cumulative injuries, surgeries, or joint injections at the time of our data collection; (4) only 53% of the cohort being clinically evaluated by their team physicians for hip and knee OA.

### 4.2. Association between Pain or Function and Clinical Knee or Hip OA

Pain might present as one of the hallmark symptoms of OA. Our research is one of few studies that has found a significant and strong association between active professional male football players with knee pain and clinical knee OA. A previous cross-sectional study amongst retired male football players found a significant association between knee pain and OA. However, there was no association between knee pain and radiographic knee OA [33].

There are only a few publications related to hip pain and clinical hip OA in active athletes, especially in the active professional male footballer. A questionnaire-based study evaluated hip and groin pain among sub-elite football players and reported that 49% complained of hip or groin pain [34]. However, this study did not utilise outcomes specifically relating to hip OA. A systematic review and meta-analysis found that 0–17% of asymptomatic athletes had clinical or radiographic hip OA compared to 2% in symptomatic athletes [35]. Our research has contrastingly found a significant and strong association between hip pain and clinical OA.

Research has found that there is a significant association between traumatic knee injuries and PROMs [36], function [37], and quality of life [38]. Our findings were contrary to these findings. We found a non-significant association between knee function and clinical knee OA in active professional male footballer. The difference in the reporting of our findings compared to the previously mentioned study’s findings may be due to (1) the different validated tools utilised to collect the information (e.g., International Knee Documentation Committee); (2) the short time interval between the injury or surgery and the conducted study; (3) the level of participation (recreational versus professional); (4) absent or poor post-injury or post-surgery rehabilitation; (5) that professional male football players are engaged in football-specific skills and other physical training (e.g., strength work and proprioceptive work) regularly as part of their professional careers. The literature has already established that exercise benefits the patient with knee OA. The recent OPTIKNEE consensus statement suggests that clinicians use credible functional PROMs to monitor and identify those at risk of developing (or who have developed) early PTOA to design a management strategy. This strategy may include specific exercise prescription (e.g., strength and proprioception) [39].

A previous cross-sectional study used validated PROMs and clinical hip OA evaluation in assessing hip function in active and retired footballers with or without clinical hip OA. They found a significant association between function and the presence of hip OA in both the active and retired professional male footballers [32]. Our study also found a significant and moderately strong association between hip function and clinical hip OA. Both studies used the same PROM (HOOS-PS) and clinical evaluation criteria (NICE criteria) for clinical hip OA. We concluded that the association between the function of the hip and the presence of clinical OA was significant due to (1) the possible presence of other hip pathology (e.g., Femoro-Acetabular Impingement Syndrome [FAI], labral tears, and hip flexor strains); (2) football being a multi-directional sport has several acute directional change movements.

### 4.3. Association between Injury or Surgery and Clinical Knee or Hip OA

There exists no comparative data from other literature as most research has focused on the association between previous injuries or surgery and the development of OA in the knee or hip of retired professional football players and not active professional male football players.

A cross-sectional study used validated PROMs and radiographic investigations to assess 1200 retired professional male footballers for the risk factors for knee OA [33]. They concluded that a football-related injury was the strongest risk factor for developing knee OA, possibly leading to knee arthroplasty later. The recent OPTIKNEE consensus statement relating to knee health after traumatic injury also suggests that PTOA has an earlier onset after a traumatic knee injury [39]. However, the literature does not define the term “earlier onset”. We found that the odds of clinical knee OA with one or more injuries sustained was 1.49 times more than the footballer that has not sustained a knee injury. We also found that the odds of clinical knee OA with one or more surgeries performed were 4.35 times more than the footballer who has not had any surgery.

There is minimal research on the association between hip injuries or surgery and clinical hip OA in the active professional male footballer. Our study suggests a non-significant association between hip injury or surgery and clinical hip OA in active professional male footballers. Potential explanations are (1) other pre-cursor pathology (e.g., FAI) may exist; (2) football-related strength and conditioning often involve hip stabilisers which may delay the clinical diagnosis of hip OA.

We opted to perform a post hoc analysis of some of the collected data that were not part of our main aims of the study. We aimed to determine if there existed an association between playing position and clinical knee or hip OA. There are no studies that investigate if an association exists between playing position and clinical knee or hip OA. We found there to exist no association between playing position and clinical knee OA and a non-significant association between playing position and clinical hip OA in the active professional male footballer. We concluded that these findings were possibly due to our cohort involving only a small percentage of the participants playing in a forward position (n = 11). A previous systematic review found that forwards may be at a higher risk of sustaining injuries compared to other outfield players [40] while goalkeepers were the least at risk for injuries. There was also a non-significant association between global health (PROMIS-GH) and clinical knee or hip OA in the active professional male footballer. The results of this analysis are potentially due to our inclusion criteria where the active professional male footballer: (1) is of a younger age; (2) is physically active more often; (3) has a different mentality to remain active as it involves their income stream; (4) has a normal BMI.

### 4.4. Study Strengths and Limitations

This is the first study to explore clinical knee and hip OA in active male professional footballers in isolation and investigates the association between (1) risk factors (injury and surgery), (2) pain, (3) knee and hip function and the presence of clinical knee or hip OA. Even if the number of participants enrolled in our study might be limited, our study achieved the minimum number of calculated sample size in order to determine significant associations.

A possible limitation is that just over half of the participants (53%) were clinically evaluated for knee and hip OA by their physicians. The data collection occurred during instances of COVID-19 restrictions and protocols, which may have impacted our data collection procedures. We did not explore reasons further, but we have assumed that these were possibly due to (1) the inability to be evaluated by their physicians due to various constraints, (2) physicians not willing to assist with data collection, (3) the inability to share further information due to internal club confidentiality agreements. Furthermore, even though the KOOS-PS and HOOS-PS have been shown to be valid tools to determine how individual function is affected by knee or hip OA, a recent meta-analysis has suggested that these tools may not adequately reflect function [41]. However, when this was published, our data collection was nearly completed and adjustments to our protocol were not feasible. We also recognise that the clinical diagnosis of OA was based on clinical evaluation and may be biased due to physician experience or not applying the NICE criteria appropriately.

### 4.5. Implications in Clinical Practice

Clinicians must have a high index of suspicion of clinical OA in the athlete who has had one or multiple knee or hip injuries or surgical interventions and has pain as a predominant symptom. The hip joint appears to present with earlier pain and dysfunction compared to the knee joint. Validated tools and clinical evaluation should be the cornerstones in the early identification of active professional male footballers with knee or hip OA. This will assist physicians in formulating an individualised management strategy to allow safe and effective continuation of participation. This management strategy can then be further developed as the player considers retirement and the long-term sequelae of OA can be managed using appropriate and effective interventions.

### 4.6. Implications for Research

Our data has identified a lack of longitudinal research related to the active professional male football player and clinical knee or hip OA. Most research has focused on the retired professional male football player and the risk and prevalence of knee and hip OA and further interventions (e.g., arthroplasty). Future research should further investigate identifying the prevalence, risk factors (including playing position), and effect on function with knee or hip OA in active professional male football players. This will assist and developing a preventative and guided approach for knee and hip OA management in the active professional male footballer.

## 5. Conclusions

There is a low prevalence of clinical knee (9.43%) or hip OA (7.55%) in the active professional male footballer. Pain in the knee or hip may be a valid symptom to predict or monitor knee or hip OA (knee, *p* = 0.033; hip, *p* = 0.005). Validated assessment tools should be utilised to identify a negative effect on function. The odds of developing clinical OA in the knee increased by 1.49 (95% CI; 0.16–19.32; *p* = 0.706) times more in those who sustained injuries than those who did not and increased by 4.35 (95% CI; 0.44–57.87; *p* = 0.150) times more in those who sustained surgeries than those who did not. The hip joint appears to present with earlier clinical signs of OA than the knee joint.

## Figures and Tables

**Table 1:** Characteristics of participants.

		*n*	%
Football characteristics	*Playing position*		
	Goalkeeper	23	22.80
	Defender	42	41.60
	Midfielder	25	24.80
	Forward	11	10.90
	*Career Level*		
	Highest national level	57	56.40
	Second highest national level	32	31.70
	Other levels	12	11.90
		Mean	SD
Demographics			
	*Age*	26.5	1.7
	*Height*	181.7	11.2
	*Weight*	79.2	8.2
	*BMI*	24.2	4.2
	*Seasons played*	7.6	2.6
Global Health	*Total PROMIS-GH for physical health*	62.65	4.6
	*Total PROMIS-GH for mental health*	50.96	8.6

% = percentage; SD = standard deviation; Age in years; height in centimetres; Weight in kilograms; BMI = body mass index; BMI in kilograms per square meter.

**Table 2:** Prevalence and association between pain and function and clinical knee and hip OA.

		n	%	95% CI	*p*-Value	Cramer’s V Value
Prevalence	*Clinical knee OA*	5	9.43	1.56–17.30		
	*Clinical hip OA*	4	7.55	0.44–14.66		
Pain	*Knee pain and clinical OA*	4	14.81		0.033 *	*0.523*
	*Hip pain and clinical OA*	4	23.53		0.005 *	*0.602*
		Mean	SD			
Function	*KOOS-PS score and clinical knee OA*	88.62	13.89		0.056	0.369
	*HOOS-PS score and clinical hip OA*	97.21	6.50		0.037 *	*0.529*

SD = standard deviation. Notes: Cramers V values are displayed where applicable. CI displayed where applicable. Total n evaluated by their physician for clinical OA = 53. Total n with knee pain = 27. Total n with hip pain = 17. * = significant *p*-value.

**Table 3:** Prevalence and association between injury or surgery and clinical knee or hip OA amongst those clinically evaluated.

	n	%	Fisher’s *p*-Value	OR of clinical OA after Injury or Surgery	OR 95% CI	OR *p*-Value
Knee injury and clinical OA	3	5.66	1	1.49	0.16–19.32	0.706
Knee surgery and clinical OA	3	5.66	0.131	4.35	0.44–57.87	0.150
Hip Injury and clinical OA	1	1.89	1	0.92	1.34–12.75	0.999
Hip surgery and clinical OA	0	0 ^^^	1	0 ^^^		

^^^ cannot be calculated due to zero values. Note: Total n evaluated by their physician for clinical OA = 53.

## Data Availability

All statistical analysis data are available as supplementary material. Raw data can be requested from the corresponding author, at reasonable request.

## Supplementary Materials

The following supporting information can be downloaded at: https://www.mdpi.com/article/10.3390/sports11070136/s1. Supplementary File SA: Designed questionnaire. Supplementary File SB: KOOS-PS. Supplementary File SC: HOOS-PS. Supplementary File SD: PROMIS-GH. STRENGTHS AND LIMTATIONS 24032023. T22066-Statistical-Analysis-Total Final.
