# Supplementary material for: Determination of the Prevalence of Knee and Hip Clinical Osteoarthritis in the Active Professional Male Footballer and Its Association with Pain, Function, Injury and Surgery
Source: Sports (Basel). 2023 Jul 18;11(7):136. doi: 10.3390/sports11070136 (PMC10385669; doi:10.3390/sports11070136)
Supplement: Supplementary file 1 [file sports-11-00136-s001.zip › STRENGTHS AND LIMTATIONS 24032023.pdf]

The strength of this study is that it is one of the first studies to explore clinical OA of knees and hips in active male professional footballers. This study also comments on the relationship between risk factors (injury and surgery), knee and hip function and player characteristics, with the development of clinical OA.

The authors note there were limitations to the study. Just over half of the participants were examined for OA knee and hip by their physicians. Considered reasons may be (1) unable to get to their physicians because of time constraints on their side or the physicians' side, (2) physicians not wanting to assist with data collection in doing the evaluation, and (3) players defaulting on appointments with physicians for evaluation. However, it still consisted of the minimum number sample size in order to determine significant relationships. Another limitation was that even though the KOOS-PS and HOOS-PS are valid tools to establish the effects of OA on function, a recent meta-analysis has suggested there are other better tools [34] for this cohort (the KOOS and HOOS with function and sports scoring subscales might have been a better tool) [35]. Another limitation was that the clinical diagnosis of OA was based on clinician evaluation and may be biased due to experience.
