# Supplementary material for: Determination of the Prevalence of Knee and Hip Clinical Osteoarthritis in the Active Professional Male Footballer and Its Association with Pain, Function, Injury and Surgery
Source: Sports (Basel). 2023 Jul 18;11(7):136. doi: 10.3390/sports11070136 (PMC10385669; doi:10.3390/sports11070136)
Supplement: Supplementary file 1 [file sports-11-00136-s001.zip › Supplementary file SA questionnaire.pdf]

Supplementary file A: Designed questionnaire

Demographics

1. What Is your age?

2. What is your weight?

3. What is your height?

4. Are you studying?

5. If you are studying, how many hours a day do you spend studying?

Football

6. What level of football do you play?

    Highest national level

    Second highest national level

    First division club

    Second division club

7. How long have you been playing football?

8. What position do you play?

Hospitalisation

9. Have you ever been admitted to hospital?

10. If you have been admitted, what was it for?

Medication usage

11. Do you use prescription pain medication for ankle pain?

12. Do you use counter over-the-counter medication for ankle pain?

13. Do you use protein supplements?

14. Do you use creatinine?

15. Do you use other multivitamins?

Injuries and surgery

16. How many injuries have you sustained to your left hip?

17. How many injuries have you sustained to your right hip?

18. How many surgeries have you had on your left hip?

19. How many surgeries have you had on your right hip?

20. How many injuries have you sustained to your left knee?

21. How many injuries have you sustained to your right knee?

22. How many surgeries have you had on your left knee?

23. How many surgeries have you had on your right knee?
