# Supplementary material for: Determination of the Prevalence of Knee and Hip Clinical Osteoarthritis in the Active Professional Male Footballer and Its Association with Pain, Function, Injury and Surgery
Source: Sports (Basel). 2023 Jul 18;11(7):136. doi: 10.3390/sports11070136 (PMC10385669; doi:10.3390/sports11070136)
Supplement: Supplementary file 1 [file sports-11-00136-s001.zip › Supplementary file SC HOOSPS.pdf]

## Supplementary file C: HOOS-PS

Hip disability and Osteoarthritis Outcome Score (HOOS) – Physical Function Shortform (HOOS-PS) English version

# HOOS-Physical Function Shortform (HOOS-PS)

Today's date:_______/________/______ Date of birth: _______/________/_______

Name: ____________________________________________________________

**INSTRUCTIONS:** This survey asks for your view about your hip. This information will help us keep track of how well you are able to perform different activities. Answer every question by ticking the appropriate box, only one box for each question. If you are unsure about how to answer a question, please give the best answer you can so that you answer all the questions.

The following questions concern your level of function in performing usual daily activities and higher level activities. For each of the following activities, please indicate the degree of difficulty you have experienced in the **last week** due to your hip problem.

1. Descending stairs

| **None** | **Mild** | **Moderate** | **Severe** | **Extreme** |
|---|---|---|---|---|
| ❑ | ❑ | ❑ | ❑ | ❑ |

2. Getting in/out of bath or shower

| **None** | **Mild** | **Moderate** | **Severe** | **Extreme** |
|---|---|---|---|---|
| ❑ | ❑ | ❑ | ❑ | ❑ |

3. Sitting

| **None** | **Mild** | **Moderate** | **Severe** | **Extreme** |
|---|---|---|---|---|
| ❑ | ❑ | ❑ | ❑ | ❑ |

4. Running

| **None** | **Mild** | **Moderate** | **Severe** | **Extreme** |
|---|---|---|---|---|
| ❑ | ❑ | ❑ | ❑ | ❑ |

5. Twisting/pivoting on your loaded leg

| **None** | **Mild** | **Moderate** | **Severe** | **Extreme** |
|---|---|---|---|---|
| ❑ | ❑ | ❑ | ❑ | ❑ |
