# Supplementary material for: Determination of the Prevalence of Knee and Hip Clinical Osteoarthritis in the Active Professional Male Footballer and Its Association with Pain, Function, Injury and Surgery
Source: Sports (Basel). 2023 Jul 18;11(7):136. doi: 10.3390/sports11070136 (PMC10385669; doi:10.3390/sports11070136)
Supplement: Supplementary file 1 [file sports-11-00136-s001.zip › Supplementary file SD PROMISGH.pdf]

Supplementary file D: PROMIS-GH

Global Health

Please respond to each question or statement by marking one box per row.

		Excellent	Very good	Good	Fair	Poor
Global01	In general, would you say your health is:	☐ 5	☐ 4	☐ 3	☐ 2	☐ 1
Global02	In general, would you say your quality of life is:...	☐ 5	☐ 4	☐ 3	☐ 2	☐ 1
Global03	In general, how would you rate your physical health?	☐ 5	☐ 4	☐ 3	☐ 2	☐ 1
Global04	In general, how would you rate your mental health, including your mood and your ability to think?	☐ 5	☐ 4	☐ 3	☐ 2	☐ 1
Global05	In general, how would you rate your satisfaction with your social activities and relationships?	☐ 5	☐ 4	☐ 3	☐ 2	☐ 1
Global09r	In general, please rate how well you carry out your usual social activities and roles. (This includes activities at home, at work and in your community, and responsibilities as a parent, child, spouse, employee, friend, etc.)......	☐ 5	☐ 4	☐ 3	☐ 2	☐ 1

		Completely	Mostly	Moderately	A little	Not at all
Global06	To what extent are you able to carry out your everyday physical activities such as walking, climbing stairs, carrying groceries, or moving a chair?...	☐ 5	☐ 4	☐ 3	☐ 2	☐ 1

© 2010-2018 PROMIS Health Organization (PHO)

In the past 7 days…

		Never	Rarely	Sometimes	Often	Always
Global10r	How often have you been bothered by emotional problems such as feeling anxious, depressed or irritable?	☐ 5	☐ 4	☐ 3	☐ 2	☐ 1

		None	Mild	Moderate	Severe	Very severe
Global08r	How would you rate your fatigue on average?	☐ 5	☐ 4	☐ 3	☐ 2	☐ 1

Global07r	How would you rate your pain on average?	☐ 0 No pain	☐ 1	☐ 2	☐ 3	☐ 4	☐ 5	☐ 6	☐ 7	☐ 8	☐ 9	☐ 10 Worst pain imaginable

© 2010-2018 PROMIS Health Organization (PHO)
