# Supplementary material for: Determination of the Prevalence of Knee and Hip Clinical Osteoarthritis in the Active Professional Male Footballer and Its Association with Pain, Function, Injury and Surgery
Source: Sports (Basel). 2023 Jul 18;11(7):136. doi: 10.3390/sports11070136 (PMC10385669; doi:10.3390/sports11070136)
Supplement: Supplementary file 1 [file sports-11-00136-s001.zip › T22066-Statistical-Analysis-Total Final.pdf]

T22066: Statistical Analysis

University of Pretoria

02 May, 2023

Contents

1 Prevalence **2**

2 Preliminary Tests **3**

2.1 Knee 4

2.1.1 Injuries and Surgeries 4

2.1.2 Injuries and OA 5

2.1.3 Surgeries and OA 6

2.2 Hip 7

2.2.1 Injuries and Surgeries 7

2.2.2 Injuries and OA 8

2.2.3 Surgeries and OA 9

2.3 Summary 10

3 Clinical OA : Other Comparisons with Variables **11**

3.1 Knee 12

3.1.1 Variable : AGE 12

3.1.2 Variable : HEIGHT 12

3.1.3 Variable : WEIGHT 12

3.1.4 Variable : POSITION 13

3.1.5 Variable : LEVEL 13

3.1.6 Variable : CAREER 13

3.1.7 Variable : HOSPITAL 13

3.1.8 Variable : KNEEPAIN 14

3.1.9 Variable : TOTALKOOSSCORE 14

3.1.10 Variable : PROMISRAWSCORE 14

3.1.11 Variable : PROMISRAWSCOREPHYSICAL 15

3.1.12 Variable : PROMISRAWSCOREMENTAL 15

3.1.13 Variable : BMI 15

3.2 Hip 16

3.2.1 Variable : AGE 16

3.2.2 Variable : HEIGHT . . . 16
3.2.3 Variable : WEIGHT . . . 16
3.2.4 Variable : POSITION . . . 17
3.2.5 Variable : LEVEL . . . 17
3.2.6 Variable : CAREER . . . 17
3.2.7 Variable : HOSPITAL . . . 17
3.2.8 Variable : HIPPAIN . . . 18
3.2.9 Variable : TOTALHOOSSCORE . . . 18
3.2.10 Variable : PROMISRAWSCORE . . . 18
3.2.11 Variable : PROMISRAWSCOREPHYSICAL . . . 19
3.2.12 Variable : PROMISRAWSCOREMENTAL . . . 19
3.2.13 Variable : BMI . . . 19

4 Summary of additional comparisons **20**
4.1 Knee . . . 20
4.2 Hip . . . 20

5 Odds Ratios **21**
5.1 Prevalence . . . 21
5.2 Knee . . . 22
5.2.1 Injuries and Surgeries . . . 22
5.2.2 Injuries and OA . . . 23
5.2.3 Surgeries and OA . . . 24
5.3 Hip . . . 25
5.3.1 Injuries and Surgeries . . . 25
5.3.2 Injuries and OA . . . 26
5.3.3 Surgeries and OA . . . 27

1 Prevalence

Here, we report the prevalence of hip and knee OA. This will include reporting the frequencies, proportions and corresponding Wald-adjusted confidence intervals for these proportions. Please note that in this report, the left and right sides are merged and we will be looking at Hip OA and Knee OA across both sides of the body.

Some assumptions that have been made across this section and all other sections regarding the OA presence are:

- If someone had OA in both hips, this would result in a value of 2 for 'totalhipoa'. This was disregarded and adjusted to be 1, which indicates that we have OA present in the hip, regardless of which side.

	Freq - No OA	Freq - OA	Proportion - No OA	Proportion - OA	Lower CI	Upper CI
OA - Knee	48	5	0.9057	0.0943	0.0156	0.1730
OA - Hip	49	4	0.9245	0.0755	0.0044	0.1466

We note a very low prevalence of OA across all groups. The highest prevalence of OA is found in the knee with a proportion of 9.43%.

To interpret these confidence intervals, I will use the prevalence of OA in the knee: With 95% confidence, the prevalence of OA in the right hip in male professional footballers ranged from 1.56% to 17.3%.

2 Preliminary Tests

In order to determine some initial relationships in the data, specifically, the relationships between OA, severe injury and surgery, some preliminary tests will be performed.

Since a small percentage of participants have had severe injuries and/or surgeries, Fisher's exact test (the non-parametric counterpart of Pearson's Chi-Squared Test), odds ratios (where applicable) and the Cramer's V measure will be used to explore the relationships between these variables. The use of Fisher's Test over the Chi-Squared test is due to the underlying assumption of the Chi-Squared test requiring a minimum count of 5 in each cell of a frequency table, which we do not have in this case.

The hypothesis for Fisher's Exact test is as follows:

$$H_0 : \text{the variables are independent and there is no relationship between the two variables}$$

vs.

$$H_a : \text{the variables are dependent and there is a relationship between the two variables}$$

If the resultant p-value of this test is less than 5%, we would reject the null hypothesis and conclude that we have a relationship between the two variables.

In addition to the Fisher's exact test, odds ratios will be calculated where applicable to obtain further statistical and numerical insights into the relationships between these variables of interest.

Cramer's V is a statistical test which is used to understand the strength of the relationship between two variables if they're categorical in nature. In this case, all our variables of interest are indeed categorical and therefore satisfy this underlying assumption. This test can be used to evaluate the relationship between two variables (as is the case here), and when there are two or more unique values per category (which is also satisfied).

Cramer's V measures the strength of associated between two variables on a scale ranging from 0 to 1, where 0 indicates no association between the variables and 1 indicates a perfect association between the two variables.

It is calculated in the following manner:

$$\text{Cramer's V} = \sqrt{\frac{\left(\frac{\chi^2}{n}\right)}{\min(c-1, r-1)}}$$

where

- χ^2 is the Chi-Square statistic
- n is the total sample size
- r is the number of rows
- c is the number of columns

The degrees of freedom are determined by: min(c-1,r-1).

The disadvantage of the Cramer's V measure is that we have no indication of specific relationships between the two variables. This prevents us from constructing a more complete argument for relationships identification between the two variables of interest.

Let P(A | B) denote the probability of an event A and a given event B. Consequently, let A^c and B^c denote the events other than A and B, respectively. The odds of A, say $Odds_{A|B}$ happening given B is given by :

$$Odds_{A|B} = \frac{P(A|B)}{P(A^c|B)} \tag{1}$$

The odds ratio, say r, of an event A for a given event is the odds of an event A occurring given event B, divided by the odds of event A occurring for event other than B . That is:

$$r = \frac{Odds_{A|B}}{Odds_{A|B^c}}. \tag{2}$$

2.1 Knee

2.1.1 Injuries and Surgeries

In order the investigate the relationship between injuries and surgeries, a summary table is initially created to show the number of participants who have had knee injuries and those who have had surgeries.

Table 2: Table summarising the no. of severe knee injuries vs. the no. of knee surgeries

	No. of Knee Surgeries				
	0	1	2	3	4
0	47	0	0	0	0
1	14	10	2	1	0
2	6	6	3	1	0
3	2	2	1	1	0
4	0	1	0	0	1
5	1	0	0	1	0

The rows represent the number of severe knee injuries with the columns representing the number of knee surgeries. Since this table is not immediately meaningful, I will remodel this to have a binary outcome to summarise those participants who have had knee injuries or knee surgeries to be labelled with 'Yes' and those who haven't been injured or had surgery are labelled with 'No'.

Table 3: Table summarising severe knee injuries vs. knee surgeries

	Knee Surgeries	
	No	Yes
No	47	0
Yes	23	30

The p-value of 0 is less than 5% and therefore we reject the null hypothesis and conclude that we have a significant relationship between the no. of knee injuries and surgeries.

The Cramer's V value was calculated to be 0.6165 . This indicates there is a strong relationship between the two variables in this scenario. Odds ratios are not applicable here due to the zero counts in the frequency table.

2.1.2 Injuries and OA

Table 4: Table summarising the no. of severe knee injuries vs. knee clinical OA

	Clinical OA	
	0	1
0	24	2
1	14	1
2	6	1
3	1	0
4	1	1
5	2	0

The rows represent the number of severe knee injuries on the knee with the columns representing the presence of clinical Knee OA. Since this table is not immediately meaningful, I will remodel this to have a binary outcome to summarise those participants who have had knee injuries or knee surgeries to be labelled with 'Yes' and those who haven't been injured or had surgery are labelled with 'No'.

Table 5: Table summarising severe knee injuries vs. knee clinical OA

	Clinical OA	
	No	Yes
No	24	2
Yes	24	3

The p-value of 1 is greater than 5% and therefore we do not reject the null hypothesis and conclude that we have no significant relationship between the no. of knee injuries and clinical OA diagnosis.

The Cramer's V value was calculated to be 0.05847 . This indicates there is a negligible relationship between the two variables in this scenario.

The odds of having OA in the knee is 1.5 times higher if you've had (1 or more) knee injuries than if you've had no knee injuries.

2.1.3 Surgeries and OA

Table 6: Table summarising the no. of severe knee surgeries vs. knee clinical OA

	Clinical OA	
	0	1
0	36	2
1	6	2
2	3	1
3	2	0
4	1	0

The rows represent the number of severe knee surgeries on the knee with the columns representing the presence of clinical Knee OA. Since this table is not immediately meaningful, I will remodel this to have a binary outcome to summarise those participants who have had knee surgeries or knee surgeries to be labelled with 'Yes' and those who haven't been injured or had surgery are labelled with 'No'.

Table 7: Table summarising severe knee surgeries vs. knee clinical OA

	Clinical OA	
	No	Yes
No	36	2
Yes	12	3

The p-value of 0.1306 is greater than 5% and therefore we do not reject the null hypothesis and conclude that we have no significant relationship between the no. of knee surgeries and clinical OA diagnosis.

The Cramer's V value was calculated to be 0.2271 . This indicates there is a moderate to weak relationship between the two variables in this scenario.

2.2 Hip

2.2.1 Injuries and Surgeries

Table 8: Table summarising the no. of severe hip injuries vs. the no. of hip surgeries

	No. of hip Surgeries		
	0	1	3
0	75	1	0
1	12	3	0
2	3	0	0
3	2	0	1
4	3	0	0

The rows represent the number of severe hip injuries on the hip with the columns representing the number of hip surgeries on the hip. Since this table is not immediately meaningful, I will remodel this to have a binary outcome to summarise those participants who have had hip injuries or hip surgeries to be labelled with 'Yes' and those who haven't been injured or had surgery are labelled with 'No'.

Table 9: Table summarising severe hip injuries vs. hip surgeries

	Hip Surgeries	
	No	Yes
No	75	1
Yes	20	4

The p-value of 0.0113 is less than 5% and therefore we reject the null hypothesis and conclude that we have a significant relationship between the no. of hip injuries and surgeries.

The Cramer's V value was calculated to be 0.3008 . This indicates there is a moderate to weak relationship between the two variables in this scenario.

2.2.2 Injuries and OA

Table 10: Table summarising the no. of severe hip injuries vs. hip clinical OA

	Clinical OA	
	0	1
0	36	3
1	8	1
2	2	0
3	1	0
4	2	0

The rows represent the number of severe hip injuries on the hip with the columns representing the the presence of clinincal Hip OA. Since this table is not immediately meaningful, I will remodel this to have a binary outcome to summarise those participants who have had hip injuries or hip surgeries to be labelled with 'Yes' and those who haven't been injured or had surgery are labelled with 'No'.

Table 11: Table summarising severe hip injuries vs. hip clinical OA

	Clinical OA	
	No	Yes
No	36	3
Yes	13	1

The p-value of 1 is greater than 5% and therefore we do not reject the null hypothesis and conclude that we have no significant relationship between the no. of hip injuries and clinical OA diagnosis.

The Cramer's V value was calculated to be 0.009171 . This indicates there is a negligible relationship between the two variables in this scenario.

2.2.3 Surgeries and OA

Table 12: Table summarising the no. of severe hip surgeries vs. hip clinical OA

	Clinical OA	
	0	1
0	47	4
1	2	0
3	0	0

The rows represent the number of severe hip surgeries on the hip with the columns representing the presence of clinical hip OA. Since this table is not immediately meaningful, I will remodel this to have a binary outcome to summarise those participants who have had hip surgeries or hip surgeries to be labelled with 'Yes' and those who haven't been injured or had surgery are labelled with 'No'.

Table 13: Table summarising severe hip surgeries vs. hip clinical OA

	Clinical OA	
	No	Yes
No	47	4
Yes	2	0

The p-value of 1 is greater than 5% and therefore we do not reject the null hypothesis and conclude that we have no significant relationship between the no. of hip surgeries and clinical OA diagnosis.

The Cramer's V value was calculated to be 0.05658 . This indicates there is a negligible relationship between the two variables in this scenario.

2.3 Summary

In this section, we summarise the results from each of the tests performed which investigated if significant relationships exist between injuries, surgeries and clinical OA in the left and right hips and knees.

Table 14: Table summarising Section results of left and right hip and knee relationships between injury, surgery and OA

Summary of Fisher's Test Results		
	Fisher's Test P-value	Result
Knee: Injury vs. Surgery	0	Yes, we have a significant relationship
Knee: Injury vs. OA	1	No, we do not have a significant relationship
Knee: Surgery vs. OA	0.1306	No, we do not have a significant relationship
Hip: Injury vs. Surgery	0.0113	Yes, we have a significant relationship
Hip: Injury vs. OA	1	No, we do not have a significant relationship
Hip: Surgery vs. OA	1	No, we do not have a significant relationship

Based on the results summary table above, we note that we have a few significant relationships. We have significance when the p-value is smaller than 5%.

Note that since we have quite a few zero counts, we cannot calculate the odds ratios since it will lead to division by zero.

3 Clinical OA : Other Comparisons with Variables

Here, we compare various other variables of interest to identify other possible relationships with the diagnosis of clinical OA. These variables are:

- Age
- Height
- Weight
- BMI (calculated from weight and height)
- Position
- Level
- Career
- Hospital
- Hippain (for the left and right hips ONLY)
- Kneepain (for the left and right knees ONLY)
- Koos Score
- Hoos Score
- Promis Physical Score
- Promis Mental Score

These variables will be compared to the left and right knee and hip for clinical OA.

Note that for numerical variables such as the age, height, weight, BMI and career, provisional groupings/intervals were created to enable a "categorical" variables which contains counts of individuals falling within these categories rather than the raw values. These groupings appear in each section and are provisional at this stage; and therefore can be changed as requested.

3.1 Knee

In this section, various associations are tested against the clinical diagnosis of OA in the knee. Please note that the raw scores for the PROMIS measure are used for consistency since many missing values were noted for this variable (and physical and mental sub-variables).

3.1.1 Variable : AGE

Table 15: Frequency table of age and OA

	0	1
22-24	3	1
25-27	35	2
28+	10	2

The p-value of 0.1712 is greater than 5% and therefore we do not reject the null hypothesis and conclude that we have no significant relationship between these two variables.

The Cramer's V value was calculated to be 0.2203 . This indicates there is a moderate to weak relationship between the two variables in this scenario.

3.1.2 Variable : HEIGHT

Table 16: Frequency table of height and OA

	0	1
159-169	6	1
170-180	12	1
181-191	21	3
192+	9	0

The p-value of 0.7712 is greater than 5% and therefore we do not reject the null hypothesis and conclude that we have no significant relationship between these two variables.

The Cramer's V value was calculated to be 0.1649 . This indicates there is a weak relationship between the two variables in this scenario.

3.1.3 Variable : WEIGHT

Table 17: Frequency table of weight and OA

	0	1
57-67	4	1
68-78	18	2
79-89	23	2
89+	3	0

The p-value of 0.7285 is greater than 5% and therefore we do not reject the null hypothesis and conclude that we have no significant relationship between these two variables.

The Cramer's V value was calculated to be 0.1396 . This indicates there is a weak relationship between the two variables in this scenario.

3.1.4 Variable : POSITION

Table 18: Frequency table of position and OA

	0	1
1	10	1
2	20	3
3	11	1
4	7	0

The p-value of 1 is greater than 5% and therefore we do not reject the null hypothesis and conclude that we have no significant relationship between these two variables.

The Cramer's V value was calculated to be 0.144 . This indicates there is a weak relationship between the two variables in this scenario.

3.1.5 Variable : LEVEL

Table 19: Frequency table of level and OA

	0	1
1	29	3
2	14	1
3	5	1

The p-value of 0.6306 is greater than 5% and therefore we do not reject the null hypothesis and conclude that we have no significant relationship between these two variables.

The Cramer's V value was calculated to be 0.09732 . This indicates there is a negligible relationship between the two variables in this scenario.

3.1.6 Variable : CAREER

Table 20: Frequency table of career and OA

	0	1
10-13	10	2
2-5	9	2
6-9	29	1

The p-value of 0.1627 is greater than 5% and therefore we do not reject the null hypothesis and conclude that we have no significant relationship between these two variables.

The Cramer's V value was calculated to be 0.239 . This indicates there is a moderate to weak relationship between the two variables in this scenario.

3.1.7 Variable : HOSPITAL

Table 21: Frequency table of hospital and OA

	0	1
0	32	4

1	16	1

The p-value of 1 is greater than 5% and therefore we do not reject the null hypothesis and conclude that we have no significant relationship between these two variables.

The Cramer's V value was calculated to be 0.0835 . This indicates there is a negligible relationship between the two variables in this scenario.

3.1.8 Variable : KNEEPAIN

Table 22: Frequency table of kneepain and OA

	0	1
0	25	1
1	13	1
2	9	1
3	1	1
4	0	1

The p-value of 0.033 is less than 5% and therefore we reject the null hypothesis and conclude that we have a significant relationship between these two variables.

The Cramer's V value was calculated to be 0.5229 . This indicates there is a moderate to relatively strong relationship between the two variables in this scenario.

3.1.9 Variable : TOTALKOOSSCORE

Table 23: Frequency table of totalkoosscore and OA

	0	1
[30,50)	1	1
[50,70)	2	1
[70-90)	16	2
[90,100)	29	1

The p-value of 0.0562 is greater than 5% and therefore we do not reject the null hypothesis and conclude that we have no significant relationship between these two variables.

The Cramer's V value was calculated to be 0.3692 . This indicates there is a moderate to weak relationship between the two variables in this scenario.

3.1.10 Variable : PROMISRAWSCORE

Table 24: Frequency table of promisrawscore and OA

	0	1
[20-30)	1	0
[30-40)	22	3
[40-50)	25	2

The p-value of 0.6942 is greater than 5% and therefore we do not reject the null hypothesis and conclude that we have no significant relationship between these two variables.

The Cramer's V value was calculated to be 0.08972 . This indicates there is a negligible relationship between the two variables in this scenario.

3.1.11 Variable : PROMISRAWSCOREPHYSICAL

Table 25: Frequency table of promisrawscorephysical and OA

	0	1
[10-20)	6	0
[20-30)	33	4
[30-40)	9	1

The p-value of 1 is greater than 5% and therefore we do not reject the null hypothesis and conclude that we have no significant relationship between these two variables.

The Cramer's V value was calculated to be 0.1158 . This indicates there is a weak relationship between the two variables in this scenario.

3.1.12 Variable : PROMISRAWSCOREMENTAL

Table 26: Frequency table of promisrawscoremental and OA

	0	1
[12-16)	15	1
[16-20]	30	4
[8-12)	3	0

The p-value of 1 is greater than 5% and therefore we do not reject the null hypothesis and conclude that we have no significant relationship between these two variables.

The Cramer's V value was calculated to be 0.1164 . This indicates there is a weak relationship between the two variables in this scenario.

3.1.13 Variable : BMI

Table 27: Frequency table of BMI and OA

	0	1
(20-22]	5	0
(22-24]	28	4
(22-26]	11	1
26+	4	0

The p-value of 1 is greater than 5% and therefore we do not reject the null hypothesis and conclude that we have no significant relationship between these two variables.

The Cramer's V value was calculated to be 0.157 . This indicates there is a weak relationship between the two variables in this scenario.

3.2 Hip

In this section, various associations are tested against the clinical diagnosis of OA in the hip.

3.2.1 Variable : AGE

Table 28: Frequency table of age and OA

	0	1
22-24	4	0
25-27	34	3
28+	11	1

The p-value of 1 is greater than 5% and therefore we do not reject the null hypothesis and conclude that we have no significant relationship between these two variables.

The Cramer's V value was calculated to be 0.08171 . This indicates there is a negligible relationship between the two variables in this scenario.

3.2.2 Variable : HEIGHT

Table 29: Frequency table of height and OA

	0	1
159-169	7	0
170-180	13	0
181-191	21	3
192+	8	1

The p-value of 0.5735 is greater than 5% and therefore we do not reject the null hypothesis and conclude that we have no significant relationship between these two variables.

The Cramer's V value was calculated to be 0.2232 . This indicates there is a moderate to weak relationship between the two variables in this scenario.

3.2.3 Variable : WEIGHT

Table 30: Frequency table of weight and OA

	0	1
57-67	5	0
68-78	19	1
79-89	22	3
89+	3	0

The p-value of 0.8053 is greater than 5% and therefore we do not reject the null hypothesis and conclude that we have no significant relationship between these two variables.

The Cramer's V value was calculated to be 0.171 . This indicates there is a weak relationship between the two variables in this scenario.

3.2.4 Variable : POSITION

Table 31: Frequency table of position and OA

	0	1
1	10	1
2	22	1
3	11	1
4	6	1

The p-value of 0.886 is greater than 5% and therefore we do not reject the null hypothesis and conclude that we have no significant relationship between these two variables.

The Cramer's V value was calculated to be 0.126 . This indicates there is a weak relationship between the two variables in this scenario.

3.2.5 Variable : LEVEL

Table 32: Frequency table of level and OA

	0	1
1	30	2
2	13	2
3	6	0

The p-value of 0.7459 is greater than 5% and therefore we do not reject the null hypothesis and conclude that we have no significant relationship between these two variables.

The Cramer's V value was calculated to be 0.1558 . This indicates there is a weak relationship between the two variables in this scenario.

3.2.6 Variable : CAREER

Table 33: Frequency table of career and OA

	0	1
10-13	11	1
2-5	11	0
6-9	27	3

The p-value of 0.8039 is greater than 5% and therefore we do not reject the null hypothesis and conclude that we have no significant relationship between these two variables.

The Cramer's V value was calculated to be 0.1484 . This indicates there is a weak relationship between the two variables in this scenario.

3.2.7 Variable : HOSPITAL

Table 34: Frequency table of hospital and OA

	0	1
0	35	1

1	14	3

The p-value of 0.0917 is greater than 5% and therefore we do not reject the null hypothesis and conclude that we have no significant relationship between these two variables.

The Cramer's V value was calculated to be 0.2627 . This indicates there is a moderate to weak relationship between the two variables in this scenario.

3.2.8 Variable : HIPPAIN

Table 35: Frequency table of hippain and OA

	0	1
0	36	0
1	11	3
2	1	0
3	0	1
4	1	0

The p-value of 0.0048 is less than 5% and therefore we reject the null hypothesis and conclude that we have a significant relationship between these two variables.

The Cramer's V value was calculated to be 0.6022 . This indicates there is a strong relationship between the two variables in this scenario.

3.2.9 Variable : TOTALHOOSSCORE

Table 36: Frequency table of totalhoosscore and OA

	0	1
[70-80)	0	1
[80-90)	3	1
90+	45	2
Missing	1	0

The p-value of 0.0365 is less than 5% and therefore we reject the null hypothesis and conclude that we have a significant relationship between these two variables.

The Cramer's V value was calculated to be 0.5286 . This indicates there is a moderate to relatively strong relationship between the two variables in this scenario.

3.2.10 Variable : PROMISRAWSCORE

Table 37: Frequency table of promisrawscore and OA

	0	1
[20-30)	1	0
[30-40)	23	2
[40-50)	25	2

The p-value of 1 is greater than 5% and therefore we do not reject the null hypothesis and conclude that we have no significant relationship between these two variables.

The Cramer's V value was calculated to be 0.04115 . This indicates there is a negligible relationship between the two variables in this scenario.

3.2.11 Variable : PROMISRAWSCOREPHYSICAL

Table 38: Frequency table of promisrawscorephysical and OA

	0	1
[10-20)	5	1
[20-30)	34	3
[30-40)	10	0

The p-value of 0.5091 is greater than 5% and therefore we do not reject the null hypothesis and conclude that we have no significant relationship between these two variables.

The Cramer's V value was calculated to be 0.1709 . This indicates there is a weak relationship between the two variables in this scenario.

3.2.12 Variable : PROMISRAWSCOREMENTAL

Table 39: Frequency table of promisrawscoremental and OA

	0	1
[12-16)	15	1
[16-20]	32	2
[8-12)	2	1

The p-value of 0.2848 is greater than 5% and therefore we do not reject the null hypothesis and conclude that we have no significant relationship between these two variables.

The Cramer's V value was calculated to be 0.2392 . This indicates there is a moderate to weak relationship between the two variables in this scenario.

3.2.13 Variable : BMI

Table 40: Frequency table of BMI and OA

	0	1
(20-22]	4	1
(22-24]	29	3
(22-26]	12	0
26+	4	0

The p-value of 0.4605 is greater than 5% and therefore we do not reject the null hypothesis and conclude that we have no significant relationship between these two variables.

The Cramer's V value was calculated to be 0.2202 . This indicates there is a moderate to weak relationship between the two variables in this scenario.

4 Summary of additional comparisons

4.1 Knee

Table 41: Table summarising Section results of knee variables vs. OA

Summary of Fisher's Test Results		
Comparison	Fisher's Test P-value	Result
Age vs. OA	0.1712	No, we do not have a significant relationship
Height vs. OA	0.7712	No, we do not have a significant relationship
Weight vs. OA	0.7285	No, we do not have a significant relationship
Position vs. OA	1	No, we do not have a significant relationship
Level vs. OA	0.6306	No, we do not have a significant relationship
Career vs. OA	0.1627	No, we do not have a significant relationship
Hospital vs. OA	1	No, we do not have a significant relationship
Knee pain vs. OA	0.033	Yes, we have a significant relationship
Total Koos Score vs. OA	0.0562	No, we do not have a significant relationship
Promis Raw Score vs. OA	0.6942	No, we do not have a significant relationship
Promis Raw Score Physical vs. OA	1	No, we do not have a significant relationship
Promis Raw Score Mental vs. OA	1	No, we do not have a significant relationship
BMI vs. OA	1	No, we do not have a significant relationship

We note that there is a relationship between OA and kneepain at a 5% level. Should be become less stringent and use a 10% level of significance, we would have significant relationship with the Total Koos score as well.

4.2 Hip

Table 42: Table summarising Section results of hip variables vs. OA

Summary of Fisher's Test Results		
Comparison	Fisher's Test P-value	Result
Age vs. OA	1	No, we do not have a significant relationship
Height vs. OA	0.5735	No, we do not have a significant relationship
Weight vs. OA	0.8053	No, we do not have a significant relationship
Position vs. OA	0.886	No, we do not have a significant relationship
Level vs. OA	0.7459	No, we do not have a significant relationship
Career vs. OA	0.8039	No, we do not have a significant relationship
Hospital vs. OA	0.0917	No, we do not have a significant relationship
Hippain vs. OA	0.0048	Yes, we have a significant relationship
Total Hoos Score vs. OA	0.0365	Yes, we have a significant relationship
Promis Raw Score vs. OA	1	No, we do not have a significant relationship
Promis Raw Score Physical vs. OA	0.5091	No, we do not have a significant relationship
Promis Raw Score Mental vs. OA	0.2848	No, we do not have a significant relationship
BMI vs. OA	0.4605	No, we do not have a significant relationship

We note a significant relationship with Hip pain and the total HOOS score with OA at a 5% level. Should be become less stringent and use a 10% level of significance, we would have a significant relationship with Hospital as well.

5 Odds Ratios

5.1 Prevalence

Here, we report the prevalence of hip and knee OA. This will include reporting the frequencies, proportions and corresponding Wald-adjusted confidence intervals for these proportions. Please note that in this report, the left and right sides are merged and we will be looking at Hip OA and Knee OA across both sides of the body.

Some assumptions that have been made across this section and all other sections regarding the OA presence are:

- If someone had OA in both hips, this would result in a value of 2 for 'totalhipoa'. This was disregarded and adjusted to be 1, which indicates that we have OA present in the hip, regardless of which side.

	Freq - No OA	Freq - OA	Proportion - No OA	Proportion - OA	Lower CI	Upper CI
OA - Knee	48	5	0.9057	0.0943	0.0156	0.1730
OA - Hip	49	4	0.9245	0.0755	0.0044	0.1466

We note a very low prevalence of OA across all groups. The highest prevalence of OA is found in the knee with a proportion of 9.43%.

To interpret these confidence intervals, I will use the prevalence of OA in the knee: With 95% confidence, the prevalence of OA in the right hip in male professional footballers ranged from 1.56% to 17.3%.

5.2 Knee

5.2.1 Injuries and Surgeries

In order the investigate the relationship between injuries and surgeries, a summary table is initially created to show the number of participants who have had knee injuries and those who have had surgeries.

Table 44: Table summarising the no. of severe knee injuries vs. the no. of knee surgeries

	No. of Knee Surgeries				
	0	1	2	3	4
0	47	0	0	0	0
1	14	10	2	1	0
2	6	6	3	1	0
3	2	2	1	1	0
4	0	1	0	0	1
5	1	0	0	1	0

The rows represent the number of severe knee injuries with the columns representing the number of knee surgeries. Since this table is not immediately meaningful, I will remodel this to have a binary outcome to summarise those participants who have had knee injuries or knee surgeries to be labelled with 'Yes' and those who haven't been injured or had surgery are labelled with 'No'.

Table 45: Table summarising severe knee injuries vs. knee surgeries

	Knee Surgeries	
	No	Yes
No	47	0
Yes	23	30

The p-value of 0 is less than 5% and therefore we reject the null hypothesis and conclude that we have a significant relationship between the no. of knee injuries and surgeries.

The Cramer's V value was calculated to be 0.6165 . This indicates there is a strong relationship between the two variables in this scenario. Odds ratios are not applicable here due to the zero counts in the frequency table.

Since we have a zero count for people have no no knee surgeries stemming from knee injuries, we cannot compute odds ratios here since it will lead to division by zero, which is undefined.

5.2.2 Injuries and OA

Table 46: Table summarising the no. of severe knee injuries vs. knee clinical OA

	Clinical OA	
	0	1
0	24	2
1	14	1
2	6	1
3	1	0
4	1	1
5	2	0

The rows represent the number of severe knee injuries on the knee with the columns representing the presence of clinical Knee OA. Since this table is not immediately meaningful, I will remodel this to have a binary outcome to summarise those participants who have had knee injuries or knee surgeries to be labelled with 'Yes' and those who haven't been injured or had surgery are labelled with 'No'.

Table 47: Table summarising severe knee injuries vs. knee clinical OA

	Clinical OA	
	No	Yes
No	24	2
Yes	24	3

The p-value of 1 is greater than 5% and therefore we do not reject the null hypothesis and conclude that we have no significant relationship between the no. of knee injuries and clinical OA diagnosis.

The Cramer's V value was calculated to be 0.05847 . This indicates there is a negligible relationship between the two variables in this scenario.

The odds of a player having OA with one or more knee injuries is 49% higher (or 1.49 times the odds) than a player with no knee injuries. The 95% confidence interval is (0.1557 ; 19.3188) for the odds ratio. The corresponding p-value is 0.7055 which is not less than 5%, which implies that this odds ratio is not statistically significant.

5.2.3 Surgeries and OA

Table 48: Table summarising the no. of severe knee surgeries vs. knee clinical OA

	Clinical OA	
	0	1
0	36	2
1	6	2
2	3	1
3	2	0
4	1	0

The rows represent the number of severe knee surgeries on the knee with the columns representing the presence of clinical Knee OA. Since this table is not immediately meaningful, I will remodel this to have a binary outcome to summarise those participants who have had knee surgeries or knee surgeries to be labelled with 'Yes' and those who haven't been injured or had surgery are labelled with 'No'.

Table 49: Table summarising severe knee surgeries vs. knee clinical OA

	Clinical OA	
	No	Yes
No	36	2
Yes	12	3

The p-value of 0.1306 is greater than 5% and therefore we do not reject the null hypothesis and conclude that we have no significant relationship between the no. of knee surgeries and clinical OA diagnosis.

The Cramer's V value was calculated to be 0.2271 . This indicates there is a moderate to weak relationship between the two variables in this scenario.

The odds of a player having OA with one or more knee surgeries is 4.348 times the odds of a player with no knee surgeries. The 95% confidence interval is (0.4433 ; 57.8692) for the odds ratio. The corresponding p-value is 0.1497 which is not less than 5%, which implies that this odds ratio is not statistically significant.

5.3 Hip

5.3.1 Injuries and Surgeries

Table 50: Table summarising the no. of severe hip injuries vs. the no. of hip surgeries

No. of hip Surgeries			
	0	1	3
0	75	1	0
1	12	3	0
2	3	0	0
3	2	0	1
4	3	0	0

The rows represent the number of severe hip injuries on the hip with the columns representing the number of hip surgeries on the hip. Since this table is not immediately meaningful, I will remodel this to have a binary outcome to summarise those participants who have had hip injuries or hip surgeries to be labelled with 'Yes' and those who haven't been injured or had surgery are labelled with 'No'.

Table 51: Table summarising severe hip injuries vs. hip surgeries

Hip Surgeries		
	No	Yes
No	75	1
Yes	20	4

The p-value of 0.0113 is less than 5% and therefore we reject the null hypothesis and conclude that we have a significant relationship between the no. of hip injuries and surgeries.

The Cramer's V value was calculated to be 0.3008 . This indicates there is a moderate to weak relationship between the two variables in this scenario.

The odds of a player having hip surgery with one or more hip injuries is 14.47 times the odds of a player with no hip injuries. The 95% confidence interval is (1.3388 ; 744.6904) for the odds ratio. The corresponding p-value is 0.0119 which is less than 5%, which implies that this odds ratio is statistically significant.

5.3.2 Injuries and OA

Table 52: Table summarising the no. of severe hip injuries vs. hip clinical OA

	Clinical OA	
	0	1
0	36	3
1	8	1
2	2	0
3	1	0
4	2	0

The rows represent the number of severe hip injuries on the hip with the columns representing the the presence of clinincal Hip OA. Since this table is not immediately meaningful, I will remodel this to have a binary outcome to summarise those participants who have had hip injuries or hip surgeries to be labelled with 'Yes' and those who haven't been injured or had surgery are labelled with 'No'.

Table 53: Table summarising severe hip injuries vs. hip clinical OA

	Clinical OA	
	No	Yes
No	36	3
Yes	13	1

The p-value of 1 is greater than 5% and therefore we do not reject the null hypothesis and conclude that we have no significant relationship between the no. of hip injuries and clinical OA diagnosis.

The Cramer's V value was calculated to be 0.009171 . This indicates there is a negligible relationship between the two variables in this scenario.

The odds of a player having Hip OA with one or more hip injuries is 0.9244 times the odds of a player with no hip injuries. This means that the odds of a player having Hip OA is lowered by 0.0756% if they have had no hip injuries, which is not intuitive. The 95% confidence interval is (0.0163 ; 12.7495) for the odds ratio. The corresponding p-value is 0.9987 which is greater than 5%, which implies that this odds ratio is NOT statistically significant.

5.3.3 Surgeries and OA

Table 54: Table summarising the no. of severe hip surgeries vs. hip clinical OA

	Clinical OA	
	0	1
0	47	4
1	2	0
3	0	0

The rows represent the number of severe hip surgeries on the hip with the columns representing the presence of clinical hip OA. Since this table is not immediately meaningful, I will remodel this to have a binary outcome to summarise those participants who have had hip surgeries or hip surgeries to be labelled with 'Yes' and those who haven't been injured or had surgery are labelled with 'No'.

Table 55: Table summarising severe hip surgeries vs. hip clinical OA

	Clinical OA	
	No	Yes
No	47	4
Yes	2	0

The p-value of 1 is greater than 5% and therefore we do not reject the null hypothesis and conclude that we have no significant relationship between the no. of hip surgeries and clinical OA diagnosis.

The Cramer's V value was calculated to be 0.05658 . This indicates there is a negligible relationship between the two variables in this scenario.

Odds ratios are not applicable here due to the zero counts in the frequency table.
